# On Diseases of the Skin—the Predominance of Certain Forms of Cutaneous Diseases

**Published:** 1841-02-13

**Authors:** C. N. Berkeley


### MEDICAL EXAMINER.

DEVOTED TO MEDICINE, SURGERY, AND THE COLLATERAL SCIENCES.

No. 7.] PHILADELPHIA, SATURDAY, FEBRUARY 13, 1841. [Vol. IV.

### ORIGINAL COMMUNICATION.

*On Diseases of the Skin—the predominance of certain forms of cutaneous diseases.* By C. N. Berkeley, M. D.

In the plan* published in the first number of the Examiner for 1841, a general view of all the diseases of the skin, with their varieties and synonymes, was given, that an opportunity might be afforded of becoming familiar with the nomenclature adopted abroad, and also that the synonymes of different authors might, as far as possible, be applied to the special diseases they represent, under the names most commonly used by English writers. Though these synonymes and varieties have been presented in deference to the opinions of those who have made cutaneous diseases the study of their lives, it will be found, we think, that many of them are unnecessary for the description of such as occur in this country, owing not so much to the diseases themselves being of less frequent prevalence, as to their presenting fewer varieties. The assertion that cutaneous diseases, though frequent in occurrence, are not various in extent among us, may seem strange to those whose attention has not been especially attracted to the subject. Eruptions are seen presenting the external appearances of pustules, papules, vesicles, &c., and consequently the impression is made that the diseases are pustular, papular, vesicular, &c., according to the existence of one or the other characteristic; and a general conclusion is at once drawn as to the relative frequency of occurrence and varieties of these affections, according to the opportunities of the practitioner for observing them. But a closer investigation will show the incorrectness of such conclusions, by demonstrating the connection and dependence of these appearances upon one another, and the absolute production of many of them as consequences of others; so that by tracing carefully back the progress of disease, and watching its development, we find cases of apparently the most opposite and irreconcileable characters all deducible from the most simple common origin. Thus in thirty cases under our own observation within a short time past presenting themselves at various stages of development, twenty-five were clearly traced to an original vesicular affection: of these only five exhibited vesicles unconnected with either pustules, papules, or scales, while twenty presented all these lesions in the most marked degree, though clearly referrible to the vesicle as an origin, the microscope plainly demonstrating the progress of their development from it. Here, then, was a series of cutaneous affections affording the appearances of at least four forms of disease when in reality, in five-sixths of them but one original type can be said properly to have existed, the other sixth having been clearly deducible from a syphilitic or venereal cause. From various facts of this kind, and from continual observation, we think ourselves justified in hazarding the assertion that the cutaneous diseases of this country, though frequent in occurrence, are not of sufficient variety to be accurately included in the arrangements of foreign writers; for though the same nomenclature in part, and to a certain extent the same divisions, will apply equally well here as abroad, when we attempt to subdivide the diseases and to trace out their minute analogies through the endless ramifications of these writers, we meet with but little success, and find ourselves lost in a maze of confusion and complication.

Our object in dwelling upon this point is to show the necessity for simplifying the classifications and nomenclatures of cutaneous diseases now in use, in order to make them applicable to those of our own country. To do this, we must retain the general divisions of pustules, papules, vesicles, &c.; but we think the numerous subdivisions they embrace as laid down in the plan before alluded to, may be with advantage in many cases omitted, and the diseases assigned to one or the other of the classes, according to their original type, merely assuming as a subdivision the terms acute and chronic. The plan already published will suffice for this purpose, as it embraces all the diseases of our country, though necessarily complicated by the numerous synonymes, subdivisions, &c., belonging to those of others. We shall refer to it as a synopsis in the course of our observations.

We proceed to a consideration of the different

* Several unimportant typographical errors occur in the paper alluded to: they are principally in the synonymes, and the reader will at once detect them. A mistake of more consequence has been made in setting up the brackets in one of the classes in the second division of diseases: the synonymes of Impetigo are correctly given, while the divisions of the two varieties are confounded together; thus Impetigo figurata should have embraced Porrigo larvalis, Tinea mucosa, and Crusta lactea, within one bracket, while Impetigo sparsa should have included Teigne and Dartre crustacée flavescente in another.

classes of cutaneous disease in the order in which they are believed most frequently to occur among us.

Excluding the exanthemata, (the eruptive fevers,) we believe it would be possible to include a very large proportion of all the cutaneous diseases of this country in the two divisions of vesicles and pustules. With the exception of a few of the tubercular forms of disease, such as Lupus, Syphilitic tubercle, Cheloides, &c. and one form of papular affection, Prurigo, (in which properly we think Lichen should be included,) nearly every case we have met with was traceable either to a vesicle or pustule as its origin. For of the squamous diseases, Psoriasis as an original specific affection seems to be of infrequent occurrence, and though numerous cases closely simulating it are continually coming under treatment, close inquiry invariably demonstrates its presence as an effect of vesicular affection generally, not as a distinct disease. Pityriasis scarcely ever is the subject of medical treatment, and Icthyosis fortunately is nearly unknown in our land. So also with regard to the diseases called Bullous; their characteristic is merely an exaggerated vesicle or blister, and we question much the propriety of assigning them a distinct class any more than we should attach a separate generic term to the minute pustule of Impetigo, and to the broad scab of Variola or of Equinia. Of the class Maculæ, Purpura, the most prominent, generally constitutes merely a symptom of a constitutional disease, not often considered among those of the skin; while the copper blotches of Syphilis and the marks of Ephelis can scarce be considered any thing more than accidental accompaniments of other diseases, and not diseases in themselves. Thus we believe a large proportion of diseases of the skin may be deduced from two original forms, the *vesicular* and the *pustular;* and by attentively considering the pathology of these, as far as it is possible to associate it with the minute structure of the skin, much may be accomplished towards the establishment at least of a rational treatment of such affections in all their varieties.*

The difficulties of establishing the special pathology of cutaneous diseases, have been already alluded to as interfering with any attempt to make it the basis of a classification; still, a general idea of the pathological condition of the skin may be obtained even without an exact knowledge of the *tissue* first affected—thus in some of the exanthemes, evidences of acute inflammation are present, as pain, redness, swelling, &c. exhibiting high irritation or inflammation of the capillaries;—so also in Impetigo, Eczema, &c. the same condition in part prevails, showing the acute nature of the diseases and their dependence on increased arterial action. On the other hand there are diseases betraying a want of action or a deficient energy in the cutaneous vessels with a depression of nervous susceptibility in the circulation at large, as in some forms of Purpura, in Chronic Rupia, Ecthyma, &c. So that within the range of diseases of exaggerated and diminished action, all those of the skin might be included. For practical purposes, such an arrangement would be sufficient as a guide to treatment, provided a correct diagnosis be drawn, and a distinction made between diseases of an *idiopathic* and those of a *symptomatic* character, or in other words, between affections of the skin proper and those indicative of constitutional derangement. But it is not necessary to confine ourselves to so general a view of the pathology of these diseases; many of them betray not merely a certain condition of excessive or diminished vascular action in the skin generally, but also its exact origin and seat: thus Acne is shown to be dependent on an abnormal state of the sebacious follicles,—Porrigo on an affection of the tissue secreting the hair,—Psoriasis on excessive action of the vessels producing the Epidermis, &c. So that we have the power absolutely of establishing the pathology of many diseases of the skin, though not to the extent of making the same a safe basis of classification. We proceed to a consideration of the pathology of vesicular diseases.

The simplest lesion presented in this form of cutaneous affections, is a little elevation of the cuticle, either acuminated, rounded or flattened, and filled with clear *serum;* this vesicle does not at first appear to be dependent on inflammation of any tissue, but upon some excitant applied directly to the surface itself, or upon sympathy between the skin and some internal organ: after continuing a short time its character is changed, the serum becomes turbid, and the surface on which it rests gives evidences of irritation or of inflammation;—does this depend on the cause originally producing the vesicle, or is it the result of the action of the contents of the vesicle on the parts subjacent? Rayer considers Eczema (the most common form of vesicular affection) dependent on some

* It would be an act of injustice not to call attention to the little work on cutaneous diseases by M. C. Martins, "Les Principes de la Methode Naturelle Appliqueés a la Classification des Maladies de la peau," in which he boldly advances the opinion that diseases of the skin may be deduced from a very few original forms; and though observation induces us to differ from M. Martins as to the exact forms which originate these diseases, still we cannot but bear testimony to the general merits of the work, no less than to the beautiful analogies it presents between the "cutaneous diseases of the vegetable world world and of man;"—besides this, the diagram that accompanies it, exhibiting the relations of diseases of the skin with one another, is ost happily conceived, and may be made of much practical value to the student. We regret that this work has never been translated and published in this country.

change in the follicles of the skin, and on inflammation of the papules; and attempts to prove this by showing that it scarcely ever occurs except on parts where the follicles exist in great numbers, in which we do not think him sustained by facts.

That the follicles are implicated in the more severe forms of Eczema and of every other vesicular disease, is true; but that *they* present the most *evident lesion* in the early stages of these affections, is not so certain; and we rather incline to the opinion that the simplest form of vesicular affection is only the result of some irritant to the surface, or of a temporary congestion of the superficial vessels causing an effusion of serum beneath the cuticle, and by its retention and pressure on the parts beneath, aided by a change in the serum itself, producing a subacute inflammation of the dermis and its tissues.* When the disease is of long standing, the follicles undoubtedly are prominently affected; but then it tends to become impetiginous, losing its characteristic *vesicle*, and assuming the *pustular* form.

This is true of all the varieties of vesicular affections, and from it chiefly results the difficulty of distinguishing them in their advanced stages from the simpler cases of pustular and papular diseases. Hence, also, the confusion of synonymes in the description of eruptions of the scalp and face; the same terms being used by different writers to express diseases of the most opposite characters.

But there is another portion of the skin as intimately concerned in these affections as the follicles; and it is by no means certain that the lesions of this are not the first in succession to the original formation of the vesicle—we mean the *papillæ*, or organs of touch. Whatever the nature of this tissue may be, whether in reality the terminations of the cutaneous nerves coming from the cerebro-spinal axis—whether an exquisitely sensitive erectile vascular tissue—or whether an independent *peripheral nervous system*, in it undoubtedly are developed most acutely the *consequences* of this class of cutaneous diseases. The irritation originally caused by the effused serum may indeed *superinduce* inflammation of the follicles, but the *effects* are developed through the papillary bodies; and though the follicles may take on consecutive disease, the consequences are only truly shown in the excessive tenderness and irritability of this *peculiar nervous system*. Thus in acne, sycosis, &c., where the affection is seated in the follicles and unattended with an effusion of serum, we do not often find much pain or irritation present; and, indeed, a majority of these cases never come under the notice of the physician at all, owing to the comparatively trifling inconvenience they produce; but in even the simplest vesicular diseases the reverse prevails; a degree of irritation, if not of acute inflammation, generally attends,—and in exact ratio with the number of vesicles, and the consequent development of the affection over the papillæ, is the degree of suffering the patient undergoes; showing, we think, that vesicular eruptions generally are more dependent on irritation of the papillæ, or nervous system of the skin, *consequent on serous effusion*, than upon inflammation or any other abnormal condition of the follicles. To sum up briefly our ideas of the pathology of vesicular diseases, excluding scabies, we think that at first there is an effusion of serum from the exhalents beneath the cuticle dependent on some irritant to the surface, on sympathy with an internal organ, or on congestion of the superficial capillaries: the serum, either by pressure or from a change in its character produces irritation of the papillæ, with consequent itching, tenderness, and pain; and lastly, the follicles become implicated, at which point we believe the characteristic vesicle is lost, and a pustule becomes the type of the disease.

As the treatment of vesicular and pustular diseases varies but little, its consideration will be postponed till we come to speak of the latter. Indeed, they resemble each other so closely in some of their varieties, that it is extremely difficult to draw the line of distinction, either in their kind or in their treatment. We therefore prefer postponing our remarks upon it till both classes of disease have been disposed of, that no more time and space may be occupied than necessary for presenting them fairly to the view of those interested in this branch of medicine.

* The effects of *serum* beneath the cuticle we see in blistered surfaces, whether from burns or vesicatories, where great pain and irritation are caused, unless the fluid is freely evacuated; hence, also, it is questionable whether most benefit or harm results from the presence of the cuticle on the blistered surface after the serum is drawn off: it is certainly useful in protecting the raw surface from the air, and as certainly injurious from the irritation it produces on the same.

